# Domestic and International Perspectives on Financing Adult Education and Training

**DOI:** 10.1093/geroni/igab046.1509

**Published:** 2021-12-17

**Authors:** Abigail Helsinger, Oksana Dikhtyar, Phyllis Cummins, Nytasia Hicks

**Affiliations:** 1 Scripps Gerontology Center, Miami University, Oxford, Ohio, United States; 2 Miami University, Oxford, Ohio, United States; 3 GRECC - STVHCS Department of Veterans Affairs, San Antonio, Texas, United States

## Abstract

Adult education and training (AET) over the life-course is necessary to participate in economic, social, and political activities in the time of globalization and technological advancement. However, little research has been done to identify mechanisms to fund AET opportunities among middle-aged and older adults from a comparative international perspective. Our study aimed to identify strategies to finance AET opportunities for middle-aged and older adults through an international lens, to help identify barriers and facilitators in effort to best support adult learners regardless of education background or socioeconomic characteristics. We carried out a descriptive qualitative study to facilitate an in-depth understanding of funding mechanisms available to adult learners in the selected countries, from the perspective of adult education and policy experts. Data were collected using semi-structured interviews with 61 international adult education experts from government agencies, non-governmental organizations, and education institutions. Our informants represented 10 countries including Australia, Canada, Germany, Italy, the Netherlands, Norway, Singapore, Sweden, the United Kingdom, and the United States. Data included at least one in-depth phone or web-based qualitative interview per informant in addition to information gathered from written materials (e.g., peer-reviewed publications and organizational reports). We identified three financing options that arose as themes: government-sponsored funding; employer-sponsored funding; and self-funding. We found that government-sponsored funding is especially important for low-skilled, low-income older adults for whom employer-sponsored or self-funding is not available. Our results have implications for lifelong AET policy changes, such as adaptations of successful AET funding programs across global communities.

